# Distinct signatures on d‐galactose‐induced aging and preventive/protective potency of two low‐dose vitamin D supplementation regimens on working memory, muscular damage, cardiac and cerebral oxidative stress, and SIRT1 and calstabin2 downregulation

**DOI:** 10.1002/fsn3.3422

**Published:** 2023-05-22

**Authors:** Sahar Salemi, Mohammad Yasin Zamanian, Lydia Giménez‐Llort, Zahra Jalali, Mehdi Mahmoodi, Maryam Golmohammadi, Ayat Kaeidi, Zahra Taghipour, Morteza Khademalhosseini, Mona Modanloo, Mohammad Reza Hajizadehi

**Affiliations:** ^1^ Department of Biochemistry, School of Medicine Rafsanjan University of Medical Sciences Rafsanjan Iran; ^2^ Neurophysiology Research Center Hamadan University of Medical Sciences Hamadan Iran; ^3^ Department of Pharmacology and Toxicology, School of Pharmacy Hamadan University of Medical Sciences Hamadan Iran; ^4^ Institute of Neuroscience & Department of Psychiatry and Forensic Medicine Universitat Autònoma de Barcelona Barcelona Spain; ^5^ Department of Clinical Biochemistry, Afzalipoor Faculty of Medicine Kerman University of Medical Sciences Kerman Iran; ^6^ School of Medicine Shahid Beheshti University of Medical Sciences Tehran Iran; ^7^ Department of Physiology, School of Medicine Rafsanjan University of Medical Sciences Rafsanjan Iran; ^8^ Department of Anatomy, School of Medicine Rafsanjan University of Medical Sciences Rafsanjan Iran; ^9^ Department of Pathology, School of Medicine Rafsanjan University of Medical Sciences Rafsanjan Iran; ^10^ Pharmaceutical Sciences Research Center Mazandaran University of Medical Sciences Sari Iran

**Keywords:** aging, calstabin2, oxidative stress, SIRT1, vitamin D

## Abstract

Chronic administration of d‐galactose (d‐gal) in rodents reproduces the overproduction of reactive oxygen species of physiological aging. The present research shows for the first time distinct signatures on d‐gal‐induced aging (500 mg/kg, 6 weeks) and the preventive and protective potential of two vitamin D (50 IU) supplementation regimens (pre‐induction and simultaneous, respectively) in two vital organs (heart and brain). d‐gal‐induced notorious alterations in working memory, a strong increase in brain malondialdehyde (MDA) oxidative levels, and strong downregulation of sirtuin 1 (SIRT1) in the heart and hippocampus and of calstabin2 in the heart. Cardiac and brain superoxide dismutase (SOD) and glutathione peroxidase (GPx) enzymatic antioxidant capacities were damaged, brain calstabin2 was downregulated, and neuropathology was observed. Heart damage also included a moderate increase in MDA levels, serologic lactate dehydrogenase (LDH), total creatine kinase (CK) activities, and histopathological alterations. The used dose of vitamin D was enough to prevent cognitive impairment, avoid muscular damage, hamper cardiac and cerebral oxidative stress, and SIRT1 and calstabin2 downregulation. Most importantly, the potencies of the two preventive schedules depended on the tissue and level of study. The pre‐induction schedule prevented d‐gal‐induced aging by 1 order of magnitude higher than simultaneous administration in all the variables studied except for SIRT1, whose strong downregulation induced by d‐gal was equally prevented by both schedules. The benefits of vitamin D for oxidative stress were stronger in the brain than in the heart. Brain MDA levels were more sensitive to damage, while SOD and GPx antioxidant enzymatic activities were in the heart. In this order, the magnitude of SOD, MDA, and GPx oxidative stress markers was sensitive to prevention. In summary, the results unveiled distinct aging induction, preventive signatures, and sensitivity of markers depending on different levels of study and tissues, which are relevant from a mechanistic view and in the design of targeted interventions.

## Graphical abstract

Heart and brain d‐gal‐induced aging and preventive potencies of two Vit D regimes were distinct. d‐gal‐induced impairment was notorious in cognition and brain MDA levels and prevented by Vit D.

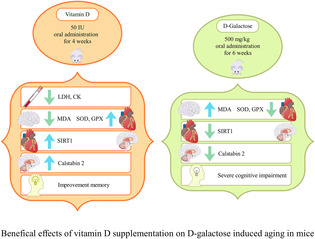

## 1 INTRODUCTION

Aging, a natural process defined as the progressive structural and functional decline in organisms with time, is a significant risk factor for vital organs such as the heart and brain (Peters, 2006; Yuan, Cruzat, et al., 2016). Various theories explain the complex and diverse number of changes associated with this process, from the molecular to the organism level (da Costa et al., 2016). Harman's free radical theory of aging proposed that the accumulation of oxidative damage to cells and tissues was associated with aging and degenerative diseases (Li et al., 2022; Pomatto & Davies, 2018). Antioxidant enzymes, including superoxide dismutase (SOD), catalase, and glutathione peroxidase (GPx), are endogenous defensive lines against oxidative damage that are useful to monitor the progress of these processes and interventions' effects (Rodriguez et al., 2004).

At the experimental level, the study of natural aging in specimens confronts several intrinsic limitations, such as individual heterogeneity, comorbidities, and survival, but also extrinsic research constraints related to costly and time‐consuming approaches which can be minimized using models for accelerated aging. A well‐accepted experimental model of oxidative stress‐related physiological aging is the long‐term treatment of mice with d‐galactose (d‐gal) (Azman & Zakaria, 2019; Hakimizadeh, Zamanian, Borisov, et al., 2022; Hakimizadeh, Zamanian, Damankhorshid, et al., 2022). Chronic exposure to this reducing sugar accelerates aging through increased generation of reactive oxygen species (ROS) oxidative stress and antioxidant enzyme downregulation (Anand et al., 2012). Besides, it impacts cognition and behavior, has few side effects, and has a high survival rate, being suitable to investigate pathways and diseases related to aging and interventions. The dose of d‐gal 500 mg/kg reproduces oxidative stress and cognitive impairment within 6 weeks (Hakimizadeh et al., 2021), while more prolonged treatment is required with lower doses (100 mg/kg) (Hao et al., 2014). Baeta‐Corral et al. (2018) have demonstrated sexual dimorphism in the behavioral responses and immuno‐endocrine status in d‐gal‐induced aging, with the male sex being particularly sensitive to d‐gal.

Conversely, studies of aging at the cellular level have also identified that the regulation of certain pathways and intracellular mechanisms can delay the aging process. This is the case of sirtuins, a family of nicotinamide adenine dinucleotide (NAD)‐dependent deacetylases (Lee et al., 2019). Among the seven mammalian members (SIRT1 to SIRT7) (Chung et al., 2010), SIRT1 stands out for playing a myriad of roles in multiple tissues and organs but mostly for its relevance in aging‐associated disorders, health span, and longevity. Key cellular processes including gene silencing, mitochondrial function and biogenesis, longevity, cellular senescence, apoptosis, and cell survival are regulated by SIRT1 (Yuan, Cruzat, et al., 2016). As a result, SIRT1 is associated with a progressive reduction in chronic diseases such as neurodegenerative disease and cardiovascular diseases, and aging (Alcendor et al., 2007; Chen et al., 2020). In a mice model of Alzheimer's disease (AD), we have previously shown that prodromal cognitive impairment is concomitant to SIRT1 cortical overexpression but hippocampal downregulation, suggesting impaired antioxidative protection to prevent or delay the underlying neuronal damage in the transgenic animals (Torres‐Lista et al., 2014). Conversely, a lentivirus vector strategy to induce hippocampal overexpression of SIRT1 prevented AD‐cognitive impairment and exerted nootropic effects in animals with normal aging, which in cell cultures were shown to be done through neurotrophic and proteostatic mechanisms (Corpas et al., 2017). Non‐pharmacological strategies such as physical exercise also restored SIRT1 downregulation (Ferrara et al., 2008).

Calcium, a ubiquitous intracellular messenger, plays a key role in a wide variety of biological functions such as cell growth, differentiation, metabolism, exocytosis, and apoptosis (Balestrini et al., 2021; Micallef et al., 2009; Zhang et al., 2021). Subcellular biochemistry points at ryanodine receptors (RYRs), which is one of the intracellular Ca^2+^ release channels in the intercellular endoplasmic/sarcoplasmic reticulum (ER/SR), which is involved in several functions in health and disease. All three mammalian receptor subtypes (RyR1, RyR2, and RyR3) (Santulli et al., 2018; Yuan, Deng, et al., 2016) are found in the brain, while the RyR2 isoform is the major in cardiac muscle (Doggrell, 2005). FK506‐binding protein 12.6 (FKBP12.6), also named calstabin2, is a subunit of the RyR2 macromolecular complex. Its role in cardiac aging makes it a therapeutic target, and its stabilization has been proposed as a new approach to sudden cardiac death (Zissimopoulos et al., 2012). Recently, calstabin2 was identified as an important regulator of spatial and emotional memory in mice (Yuan et al., 2014).

Healthy nutrition is considered one first‐choice intervention among the different lifestyle strategies to decrease or hamper the risk of age‐related diseases (Cheng et al., 2010). Antioxidants from fruits, fungi, vitamins, and herbal extracts provide resistance against oxidative stress and ROS by increasing endogenous antioxidant defense enzymes. Also, these substances decreased the risk of heart and neurological disease (Ding et al., 2016; Milisav et al., 2018; Rusu et al., 2020). Essential micronutrients such as vitamin D are known to play an important role at different life cycle stages, especially in those with specific needs such as aging (Latimer et al., 2014; Wimalawansa, 2019). Vitamin D is a fat‐soluble vitamin naturally present in a few foods and dietary supplements under two main forms, D2 (ergocalciferol) and D3 (cholecalciferol) (Sahota, 2014). Endogenous vitamin D is synthesized in the skin triggered by sunlight's ultraviolet (UV) rays and modulated by multiple factors, including diet, dietary supplements, and skin color (Latic & Erben, 2020). Besides its well‐known promotion of calcium absorption in the gut, bone growth, mineralization and remodeling, and the prevention of hypocalcemic tetany and osteoporosis, vitamin D has many other roles at the cellular level (Morris et al., 2012). It uptakes intracellular calcium and its re‐uptake into the sarcoplasmic reticulum and modulates the transcription of hundreds of genes encoding proteins that regulate cell proliferation, differentiation, and apoptosis (Bivona et al., 2019; Filgueiras et al., 2020). Many tissues have vitamin D receptors, and the deficiency of vitamin D is associated with a broad range of chronic conditions including increased oxidative stress (Filgueiras et al., 2020), cardiovascular diseases, hypertension (Latic & Erben, 2020), cancer, and neurological diseases (Morello et al., 2018). Conversely, these diseases can be improved with vitamin D supplementation through its antioxidant activity.

This study aimed to perform, for the first time, a comparative analysis of the effects of vitamin D to prevent and counteract d‐gal‐induced aging in mice. To this end, the effects of simultaneous chronic administration of vitamin D and d‐gal and those when vitamin D was administered starting 4 weeks pre‐induction of aging were compared at different levels of study, from behavioral effects on cognitive function, histopathological effects on heart tissue, peripheral (serologic) markers of muscle damage, oxidative stress‐related indicators, as well as SIRT1 and calstabin2 expression in the brain and heart.

## 2 MATERIALS AND METHODS

### 2.1 Animals and treatments

Forty‐two 2‐ to 3‐month‐old male NMRI (Naval Medical Research Institute) mice (30–35 g) were housed in Plexiglas cages (7 mice per cage) at the Animal House of the Rafsanjan University of Medical Sciences. They were kept at constant temperature (23 ± 2°C) and humidity (60%), and on a 12‐h light/dark cycle (lights on at 8:00 a.m.), with free access to food (standard pellet chow; Pars Dam, Tehran, Iran) and water.

Seven days after arrival, the animals were randomly divided into six experimental groups (7 animals/group). The control group received standard drinking water (10 mL/kg) (Hakimizadeh et al., 2023), while all the other groups received d‐gal (Sigma‐Aldrich, Darmstadt, Germany) freshly dissolved in drinking water (10 mL/kg) for oral administration at a dose of 500 mg/kg/day, for 6 weeks (Hakimizadeh et al., 2021). Vitamin D (Vit D, Sigma Aldrich, USA) was dissolved in propylene glycol as a vehicle (Sigma Aldrich, USA). The treatment groups received subcutaneous injections of vehicle or vitamin D at a dose of 50 IU starting 4 weeks before the induction of aging with d‐gal (pre‐d‐gal) or simultaneously (Mehrabadi & Sadr, 2020).

The Guide for the Care and Use of Laboratory Animals (Institute for Laboratory Animal Research, National Research Council, Washington, DC, National Academy Press, no. 85–23, revised 1996) was followed. The Animal Ethics Committee of the Rafsanjan University of Medical Sciences approved this study protocol (Approval ID: IR.RUMS.REC.1399.171).

### 2.2 Cognitive function

Y‐maze was performed at the end of the treatments. Animals were acclimated 1 h before the behavioral test, which was performed in the morning between 9.00 and 12.00 a.m. by a blinded observer. The maze was cleaned with diluted ethanol (5%) between each run.

The Y‐maze was used to evaluate working memory. This maze consisted of three arms (15 × 30 × 40 cm with equal angles between arms). Briefly, the animals were put in the maze center and observed for 8 min with a digital camera. Correct spontaneous alternation of arms, defined as not revisiting the same arm twice before visiting another, was measured (Hughes, 2004). The percentage of correct alternations was calculated as the index: the number of alternations/total arm visits (minus 2) × 100.

### 2.3 Harvesting and sample preparation

Twenty‐four hours after behavioral tests (between 12.00 a.m. and 3.00 p.m.) mice of each group were euthanized. Immediately after, a blood sample was collected from the corner of the eye in glass assay tubes without anticoagulant. The serum was centrifuged at 2213 × g for 10 min and stored at −80°C until a biochemical assay was prepared for blood assay. Fresh brain and heart biopsy specimens were immediately removed and longitudinally divided into two parts. One was fixed in 4% paraformaldehyde (pH 7.4) and embedded with paraffin for histological assessment, and the other was immediately frozen with liquid nitrogen and stored at −80°C for biochemical analysis. In the case of the brain, the hippocampus was previously dissected.

### 2.4 Cardiac and brain histopathological study

Heart and brain biopsy specimens were sectioned (5 μm). Then, the sections were deparaffinized with 100% xylene and rehydrated with gradient (100%–70%) ethanol, before staining with hematoxylin and eosin (H&E). The image was captured with Olympus dp25.

### 2.5 Muscle damage—serologic assay

The creatine kinase (total CK) and lactate dehydrogenase (LDH), used as indicators of muscular tissue damage (Withee et al., 2017), were evaluated by an automatic biochemical analyzer, and the results were expressed as mg/dL.

### 2.6 Oxidative stress evaluation

Samples were homogenized (1/10 *w/v*) in ice‐cold PBS buffer (100 mM, pH 7.4), centrifuged at 4427× *g* for 20 min, and the supernatant was collected and stored at −80°C for biochemical analysis. The lipid peroxidation was measured with malondialdehyde (MDA) levels by a commercially available kit (ZellBio, Lonsee, Germany; Catalog Number: ZB‐MDA‐96A) according to the manufacturer's protocol (the detection limit: 0.1 μM and the detection range: 0.78–50 μM). SOD activity was measured with a commercially available kit (ZellBio, Germany; Catalog Number: ZB‐SOD‐96A), according to the manufacturer's protocol (the detection limit: 1 U/mL and the detection range: 5–100 U/mL). GPx activity was evaluated by a commercially available kit (ZellBio, Germany; Catalog Number: ZB‐GPX‐96A), according to the manufacturer's protocol (the detection limit: 5 U/mL and the detection range: 20–500 U/mL). The light absorption was read by the ELISA Microplate Reader (Rayto, Shenzhen Guangdong, China). All samples were analyzed in duplicate, and the results were presented as a percentage of the control.

### 2.7 Real‐time polymerase chain reaction (quantitative polymerase chain reaction (qPCR))

The transcription level of SIRT1 and calstabin2 was assessed by real‐time qPCR technique. Briefly, heart and hippocampus frozen tissues were retrieved and homogenized. Total RNA was extracted and the complementary DNA (cDNA) was synthesized using the parstous kit according to the manufacturer's instructions. Both concentration and purity were analyzed by a nanodrop DeNovix (absorbance ratio at 260/280 nm).

Quantitative gene expression analysis was conducted by application of SYBR Green I Master Mix PCR (Gene Bio, Korea) in a qPCR technique with ABI Step One plus TM qPCR system (Applied Biosystem, USA) in the presence of specific primer and was analyzed according to the 2^−ΔΔCt^ method. Β‐actin was used as a housekeeping gene (Table 1).

**TABLE 1:** Primer sequences used in this study

Gene description	Primer	Sequence (5′ → 3′)
Calstabin2	Forward: Reverse:	TCAGAATTGGCAAACAGGAAGTC TGAGCAGCTCCACGTCAAAG
SIRT1	Forward: Reverse:	TCTGAAAGTGAGACCAGTAGC ATGAAGAGGTGTTGGTGGCA
β‐Actin	Forward: Reverse:	GCGAGACCCCACTAACATCA ATGAGCCCTTCCACAATGCC

### 2.8 Statistical analysis

Statistical analysis was done by GraphPad Prism software version 9.3.1 for Windows (San Diego, California, USA). Data were presented as mean ± standard error of the mean (SEM). The differences between the experimental groups were analyzed by one‐way analysis of variance (ANOVA), followed by the Tukey post hoc test. Differences were considered statistically significant when *p* < .05.

## 3 RESULTS

### 3.1 Impaired working memory induced by d‐gal was prevented by vitamin D more efficiently when animals were pre‐treated

**FIGURE 1:**
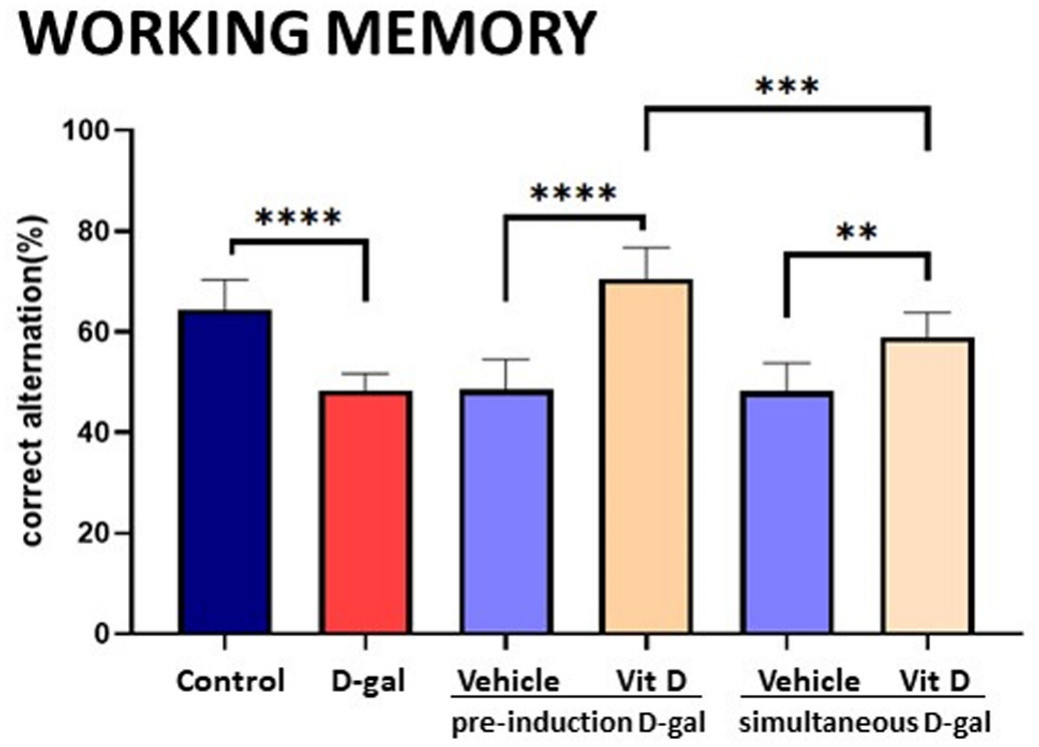
Cognitive effects—d‐gal impaired working memory in the Y maze but was prevented in groups treated with vitamin D, mainly when the animals were pre‐treated. Values are mean ± SEM (*n* = 7 in each group). ****p < .0001, ***p < .001, and ** *p* < .01 versus the group indicated.

The Y‐maze test was used to assess working memory in the different experimental groups (Figure 1). The percentage of correct alternations was significantly decreased in the d‐gal‐treated group and those pre‐treated or treated with a vehicle. In the two groups receiving vitamin D, either when the treatment started 4 weeks before the d‐gal treatment or simultaneously, the effects of d‐gal on memory were reversed (*p* < .0001 and *p* < .01, respectively). Statistically significant differences were also observed in the working memory between both vitamin D groups. Thus, when the administration of vitamin D started before d‐gal treatment, the working memory of the animals was improved in comparison to those starting the vitamin D treatment concomitantly to D‐gal (*p* < .001).

### 3.2 d‐gal increased LDH and CK levels in the serum and Vit D was more effective in CK than LDH

**FIGURE 2:**
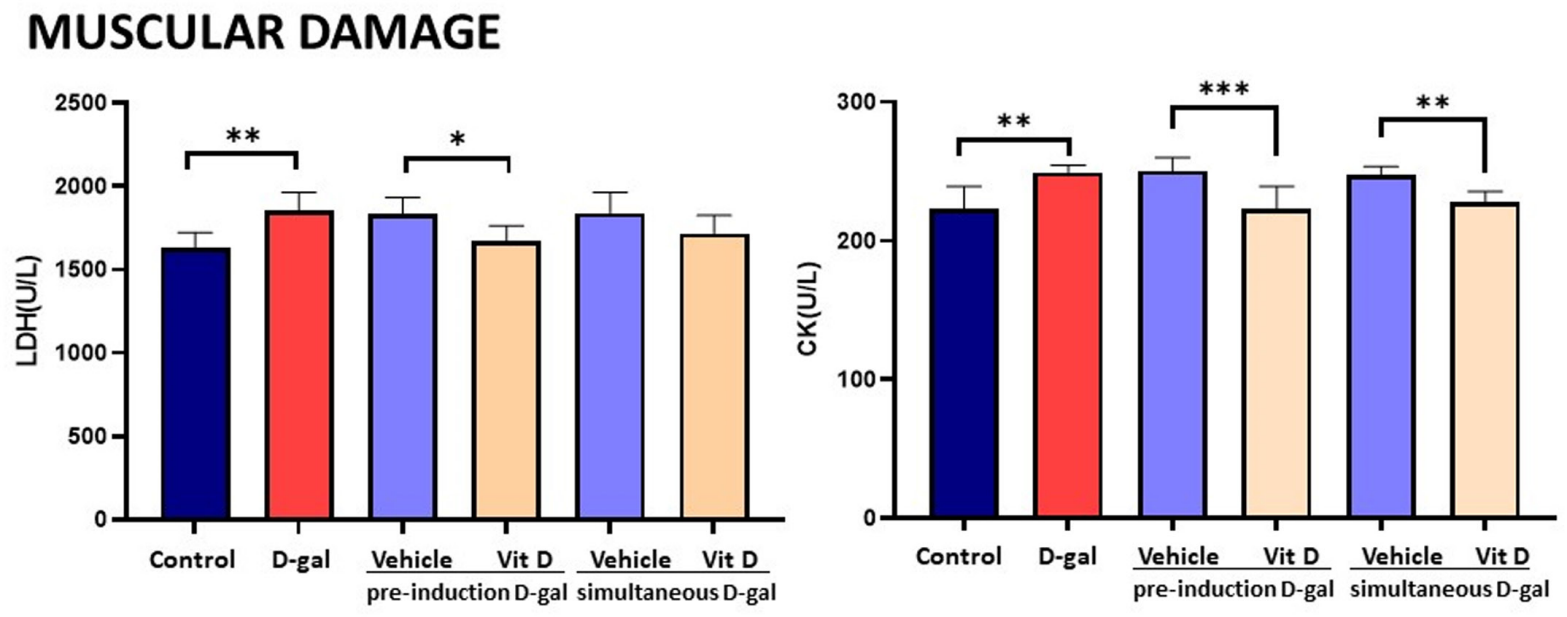
Muscular damage—d‐gal increased LDH and CK levels in the serum. The elevated levels of LDH were prevented by pre‐treatment with vitamin D; the elevated levels of CK were prevented with both treatments, but pre‐treatment was more effective. Values are mean ± SEM (*n* = 7 in each group). ***p < .001, **p < .01, and *p < .05 versus the indicated group.

The serum levels of LDH and CK are considered a marker of muscle damage. d‐gal‐treated mice that received a vehicle significantly showed increased LDH and CK (Figure 2) concentration in comparison with the control group. Vitamin D, either started to be administered before or concomitantly to d‐gal, was able to restore the levels of CK (*p* < .001 and *p* < .05, respectively), whereas LDH levels were only restored in animals for which vitamin D was started before the induction of aging (*p* < .01 vs. d‐gal group).

### 3.3 d‐gal‐induced histopathological changes in the brain but they were prevented and slightly counteracted by vitamin D

**FIGURE 3:**
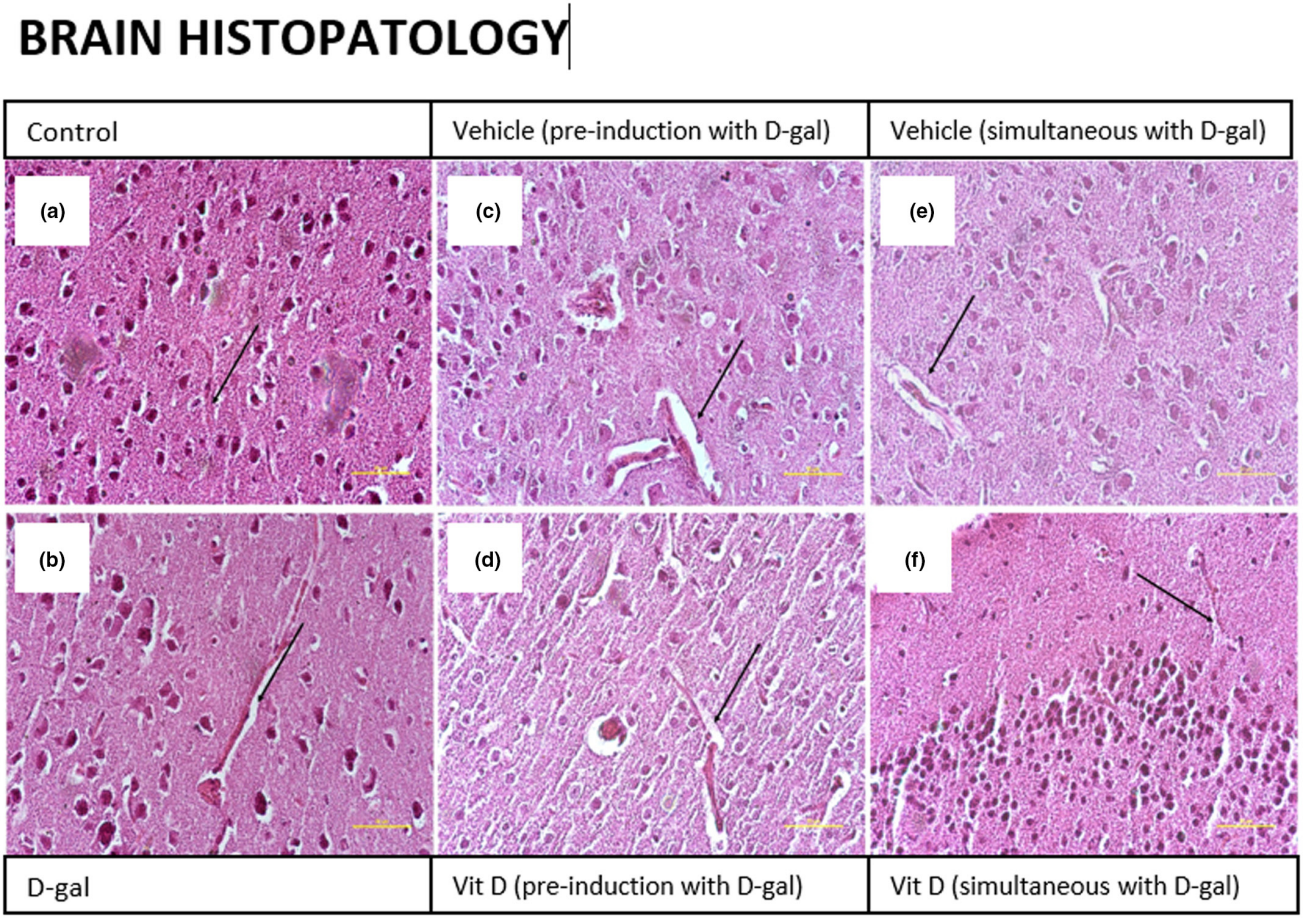
Representative illustrations of the effect of d‐gal on brain tissue and the effect of vitamin D. Control (a), d‐gal (b), vehicle pre‐induction (c), vitamin D pre‐induction (d), vehicle simultaneous with d‐gal treatment (e), and vitamin D simultaneous with d‐gal treatment (f). Arrows indicate altered and large vascular space in brain tissue.

H&E staining in brain tissue sections of d‐gal‐treated mice and both vehicle groups showed altered and larger vascular space in comparison with the control group. In contrast, the administration of vitamin D prevented these pathological changes in comparison with the d‐gal group. No difference was observed between the two vitamin D recipient groups (d‐gal pre‐treated and d‐gal simultaneous‐treated) (Figure 3).

### 3.4 Histopathological changes in the heart

**FIGURE 4:**
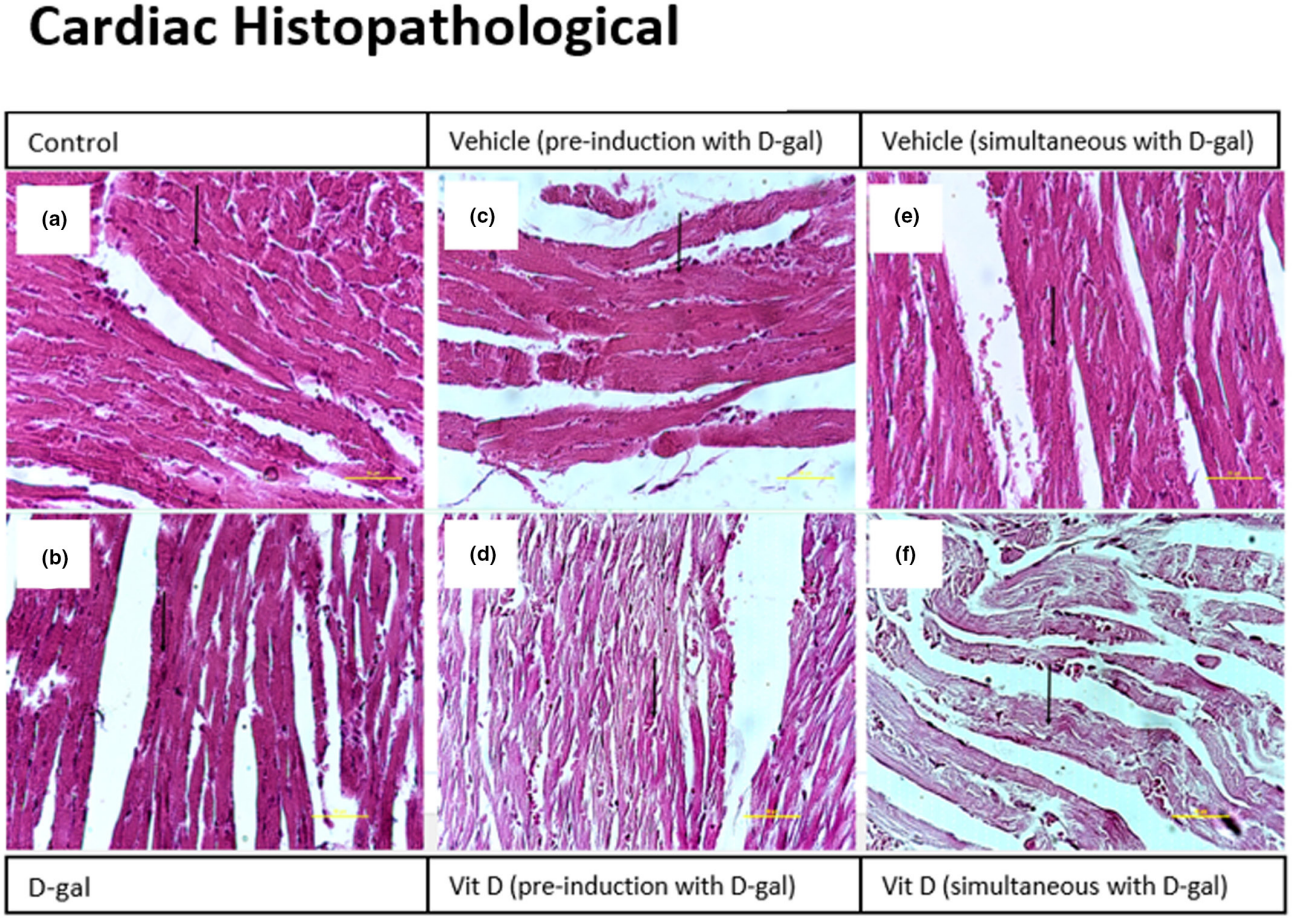
Representative illustrations of the effect of d‐gal on heart tissue and the effect of vitamin D. Control (a), d‐gal (b), vehicle pre‐induction (c), vitamin D pre‐induction (d), vehicle simultaneous with d‐gal treatment (e), and vitamin D simultaneous with d‐gal treatment (f). Arrows indicate histopathological alterations.

No difference was observed between H&E staining in the heart tissue of the study groups (Figure 4).

### 3.5 D‐gal increased markers of oxidative stress but its effects were prevented and counteracted by Vit D

**FIGURE 5:**
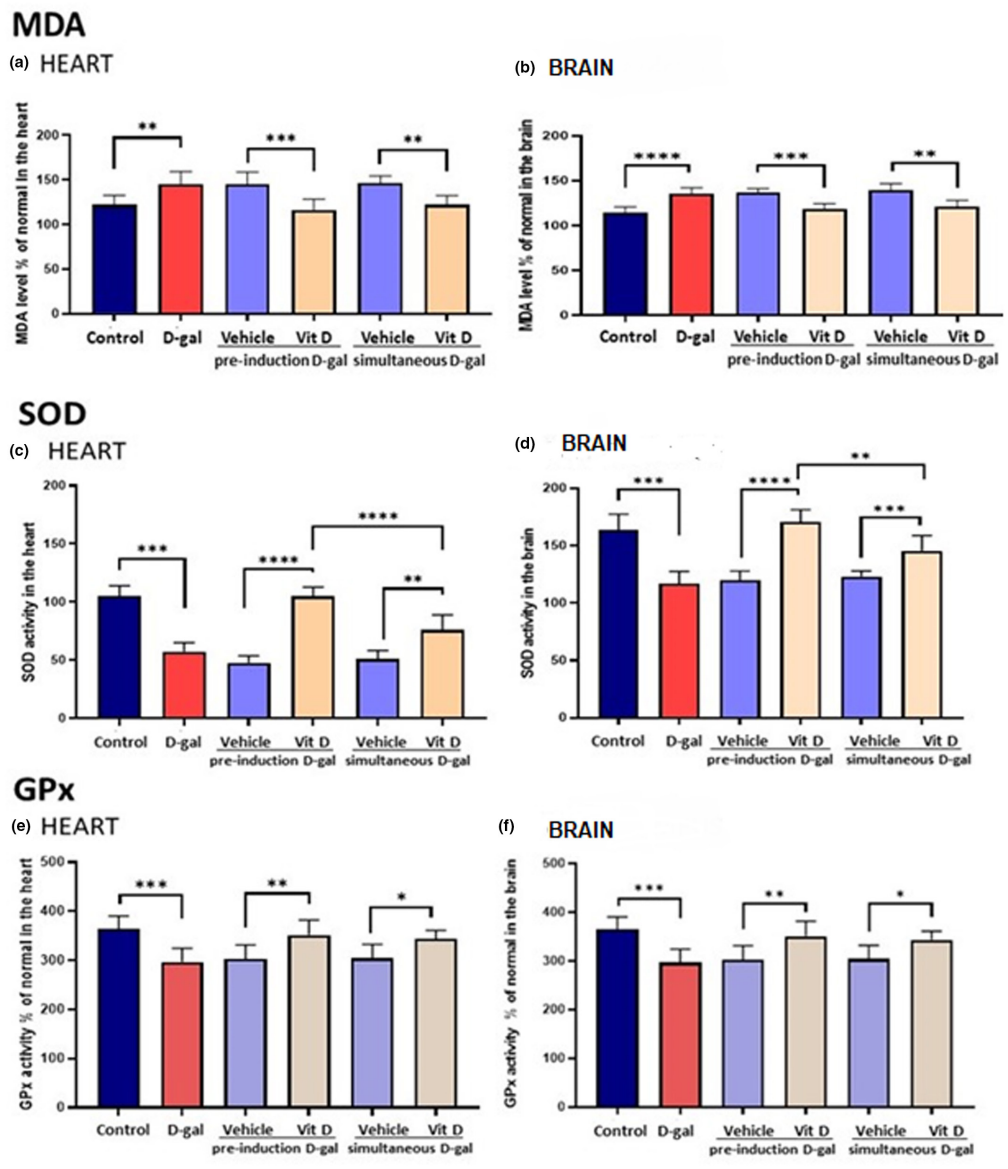
Oxidative stress—MDA levels and SOD and GPx enzymatic activities. (a) MDA levels in the heart; (b) MDA levels in the brain; (c) SOD activity in the heart; (d) SOD activity in the brain; (e) GPx activity in the heart; and (f) GPx activity in the brain. Values are mean ± SEM (*n* = 7 in each group). ***p < .001, **p < .01, and *p < .05 vs. respective control group.

Oxidative stress was measured by MDA levels (Figure 5a,b) as well as SOD (Figure 5c,d) and GPx (Figure 5e,f) enzymatic activities in heart and brain homogenates.

The MDA levels in d‐gal‐treated mice and d‐gal‐treated mice receiving vehicle were found to increase in both organs, with increases being higher in the brain (*p* < .0001) than in the heart (*p* < .01). In both cases, these increases were prevented in the groups receiving vitamin D, with higher efficiency in the pre‐treatment group (*p* < .0001) than when vitamin D was simultaneously administered with d‐gal (*p* < .01).

The SOD and GPx activities in the heart and brain of d‐gal‐treated mice and d‐gal‐treated mice receiving a vehicle were found significantly decreased compared to the control group (*p* < .001). Vitamin D prevented the decrease in both organs, with higher efficiency when vitamin D was administered before the d‐gal treatment.

### 3.6 d‐gal downregulated SIRT1 and calstabin2 expression but the effects were prevented and counteracted by vitamin D

**FIGURE 6:**
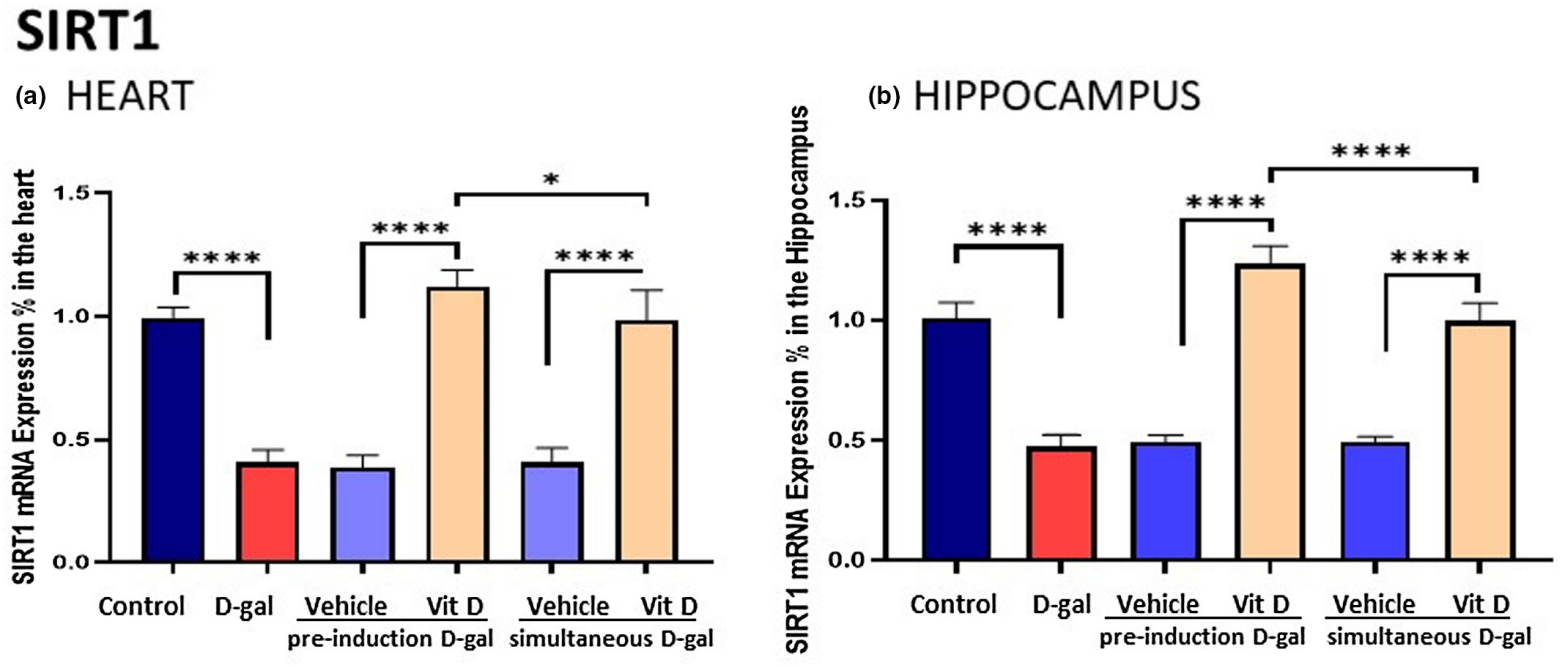
SIRT1—d‐gal decreased SIRT1 mRNA expression in the heart and hippocampus but they were prevented by vitamin D, more efficiently in the pre‐treatment schedule, mostly in the hippocampus. Values are mean ± SEM (*n* = 7 in each group). ***p < .001, **p < .01, and *p < .05 vs. respective control group.

**FIGURE 7:**
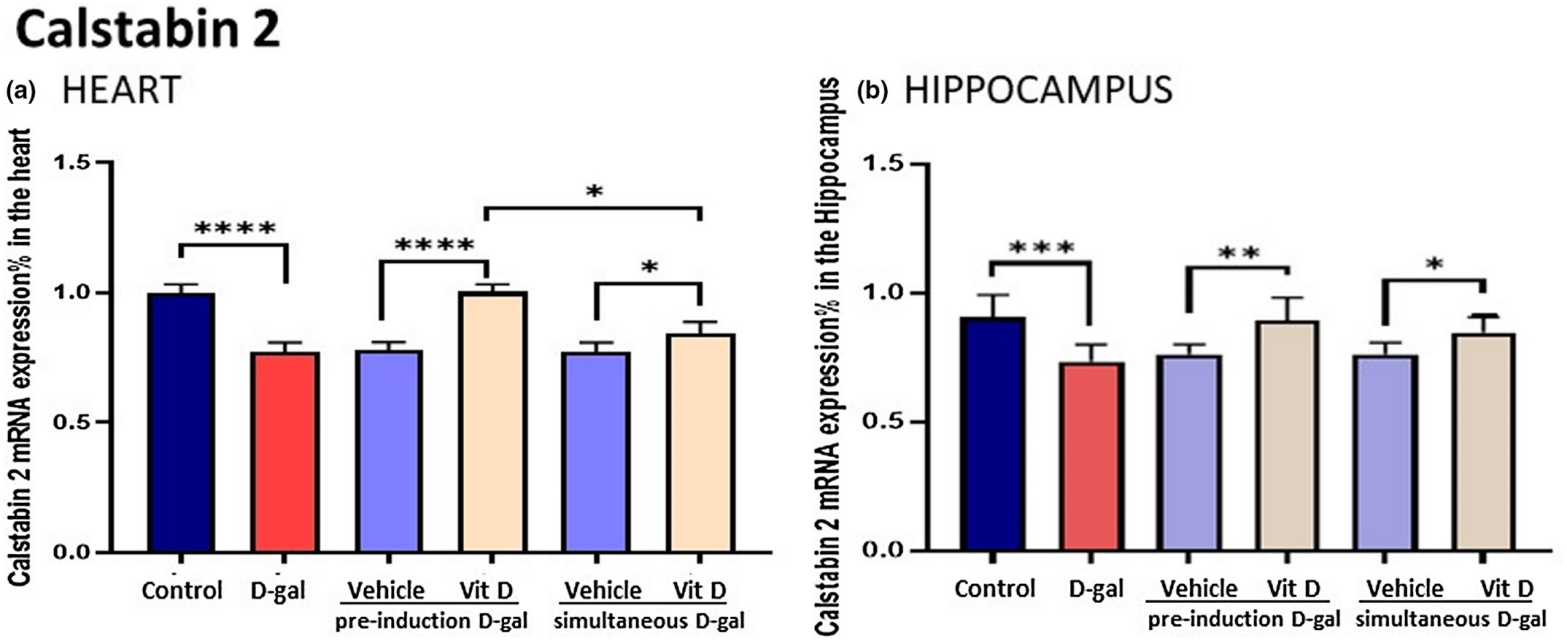
Calstabin2—d‐gal decreased calstabin2 mRNA expression in the heart and hippocampus but they were prevented by vitamin D, more efficiently in the pre‐treatment schedule, mostly in the heart. Values are mean ± SEM (*n* = 7 in each group). ***p < .001, **p < .01, and *p < .05 versus respective control group.

SIRT1 mRNA expression (Figure 6) in the d‐gal‐treated group that received propylene glycol significantly decreased in the heart and hippocampus tissues compared to the control group (*p* < .0001). In contrast, the vitamin D‐receiving groups increased SIRT1 mRNA expression in the heart and hippocampus tissues compared to the d‐gal group (*p* < 0.0001). Additionally, we observed a significant difference between SIRT1 mRNA expression in vitamin D‐receiving groups d‐gal pre‐treated and d‐gal simultaneous‐treated (heart, *p* < .05; hippocampus, *p* < .0001). Calstabin2 mRNA expression in the d‐gal‐treated group receiving propylene glycol significantly decreased in the heart and hippocampus tissues compared to the control group (*p* < .0001). In contrast, vitamin D‐receiving groups increased calstabin2 mRNA expression in the heart and hippocampus tissues compared to the d‐gal group (*p* < .0001). Also, we observed a significant difference between calstabin2 mRNA expression in vitamin D‐receiving groups d‐gal pre‐treated and d‐gal simultaneously treated (heart, *p* < .05; hippocampus, *p* < .05) (Figure 7).

## 4 DISCUSSION

This study shows, for the first time, distinct signatures of the effects of d‐gal‐induced aging and the preventive/protective potential of two vitamin D supplementation regimens at five levels of study and two vital organs, the brain and the heart. The effects of vitamin D preventing and protecting the increase in serum levels of LDH and CK, indicators of muscle damage, in d‐gal‐treated mice are also reported for the first time.

Compared to control animals, notorious alterations induced by d‐gal were shown as severe cognitive impairment, a strong increase in brain MDA oxidative levels, strong downregulation of SIRT1 in the heart and hippocampus, and of calstabin2 in the heart. In a second order of magnitude, the antioxidant capacity measured by SOD and GPX in the heart and the brain was also damaged, calstabin2 downregulated in the hippocampus, and brain histopathological alterations. At a third level, damage in the heart was shown as a moderate increase in MDA levels, LDH, and CK activities in serum. On the other hand, the beneficial effects of two vitamin D supplementation schedules, pre‐induction and simultaneous to d‐gal‐induced aging, showed that vitamin D prevented and protected the deterioration of working memory, avoided muscular damage as measured by LDH and CK, and hampered the cardiac and cerebral oxidative stress as measured by MDA levels, SOD and GPX activities, as well as SIRT1 and calstabin2 downregulation.

As expected, the pre‐induction schedule notoriously prevented the effects of d‐gal, while the statistical differences of the protective effect elicited with the simultaneous administration were 1 order of magnitude lower than those achieved with the pre‐induction. This was observed in all the variables studied, except for SIRT1, whose strong downregulation induced by d‐gal was equally effective with both schedules, indicating that SIRT1 was more sensitive/responsive to the mechanistic effects of vitamin D. Similarly, the benefits of d‐gal on oxidative stress were stronger in the brain than the heart. In the brain, MDA levels were more sensitive/responsive to damage than antioxidant enzymatic activities, while in the heart, it was the opposite, with higher magnitudes of reduction of SOD and GPx. The benefits of vitamin D also showed distinct sensitivity to stress markers, with the magnitude of SOD, MDA, and GPx showing prevention/protection in this order.

Chronic d‐gal administration reproduces the increase in brain oxidative stress underlying age‐related decline in cognitive function and the effects of aging on functional and structural brain connectivity. This model has been extensively used to study this critical cellular phenomenon and the effects of different kinds of interventions. As shown in the current study, working memory was severely affected in the groups of animals treated with d‐gal with/without a vehicle, as measured by the spontaneous alternation in the Y maze. The results confirm previous work showing that oxidative injury caused by d‐gal induces cognitive impairment, which is prominent after short chronic treatment with the high dose of 500 mg/kg (Hakimizadeh et al., 2021; Rehman et al., 2017) as compared to other doses. The increase in the antioxidant system's capacity using antioxidant agents and compounds is known to improve impaired cognition in aging humans and animals (Kaviani et al., 2017). Critical reviews in food science and nutrition in groups of interest such as children and adolescents also refer to the vitamin D status and its impact on their cognitive function through antioxidant signaling and the inhibition of the inflammatory process (Constantin et al., 2017). A recent report by Ali et al. showed for the first time the potential antioxidant effects of a higher dose of vitamin D (100 μg/kg, three times a week for 4 weeks), preventing the oxidative effect of a low dose (120 mg/kg, 8 weeks) of d‐gal on memory investigated through Morris water maze and Y maze in adult albino male mice. Their work also demonstrated that vitamin D exerted neuroprotection via SIRT1/nrf‐2/ NF‐kB signaling pathways (Ali et al., 2021).

In the current study, we provide the first evidence that a low dose of 50 IU vitamin D is enough to not only prevent but also protect the severe memory impairment caused by a high dose (500 mg/kg, oral administration, 6 weeks) of d‐gal‐induced aging, despite stronger effects were obtained when the treatment started 4 weeks before the administration of d‐gal. On the other hand, d‐gal‐induced brain aging in rodents has been previously reported to result in not only increased irregular brain tissue interstitial space (Chen et al., 2019) but also a lifestyle intervention such as nutrition with antioxidants augmented attenuated brain tissue damage (Chen et al., 2018). In agreement, using the histological level of study, the present work showed that d‐gal‐induced aging resulted in increased large vascular space in brain tissue. More importantly, the study showed the prevention of brain damage caused by d‐gal by vitamin D. Since antioxidants maintain the structure of the heart in d‐gal‐treated mice through decreased ROS and oxidative stress (Ma et al., 2021), the sequent analysis investigated the oxidative stress status and the levels of SIRT1 and calstabin2 expression in the different treatment groups.

d‐gal‐induced muscle injury is a model for aging research. The increase in ROS induced by d‐gal results in increased LDH and CK levels in serum that reflect the oxidant damage in tissues and at the myocardial level (Chen et al., 2017; He et al., 2019; Ma et al., 2021). In agreement, here we report that d‐gal increased LDH and CK levels in serum. Several works have shown that vitamin D has a direct regulatory role in skeletal muscle function, and its deficiency is associated with oxidative stress, mitochondrial function, and the development of skeletal muscle atrophy (Dzik & Kaczor, 2019). Vitamin D treatment protects against and reverses oxidative stress‐induced muscle proteolysis. Vitamin D was accompanied by lower LDH and CK levels (Qian et al., 2019). Our results provide further evidence that vitamin D prevented the increase in LDH and CK concentration induced by d‐gal. Besides, when vitamin D was administered in the pre‐induction schedule, the preventive effect on CK concentration was stronger than the protective effect achieved with the simultaneous administration. It could be concluded that vitamin D exerts a preventive and protective role in ROS damage to muscular tissue.

Many studies have reported decreased endogenous antioxidant defense, such as SOD, CAT, GSH, and GPx, as well as increased MDA levels in the heart, brain, and serum in d‐gal‐treated mice reflecting the cell damage arising from ROS (Li et al., 2016; Liu et al., 2020). As well, here, our results showed that d‐gal increased MDA levels and decreased SOD and GPx activity in the heart and brain. Concerning vitamin D, Jeremy et al. (2019) showed that it can delay testicular aging through the decrease in oxidative stress and the improvement of cellular antioxidant defense. In agreement, the present work shows that the two regimens of a low dose of vitamin D could decrease MDA levels and increase SOD and GPx activity, thus preventing and counteracting d‐gal‐induced effects. More importantly, our results demonstrate distinct signatures for vitamin D's preventive and protective effects in delaying heart and brain d‐gal‐induced aging. In agreement with our precedent work assessing different preventive/protective drugs (Fatemi et al., 2018; Kaviani et al., 2017), the prevention/protection of cognitive performance in mice treated by d‐gal exerted by vitamin D was concurrent with the storage of SOD and GPx enzymatic activity and decreasing the level of MDA.

Several studies showed that SIRT1 is low in aging (Kwon et al., 2017). SIRT1 is a redox‐sensitive protein that regulates various biological processes such as oxidative stress, apoptosis, and aging (Hsu et al., 2008). Generally, sustaining SIRT1 levels protects endothelial cells, the heart (Hsu et al., 2008), and the brain (Khan et al., 2019), and also against age‐related diseases. In agreement with previous results on chronic administration of d‐gal or increased oxidative stress decreases the expression of SIRT1 (Chen et al., 2020), our results showed downregulation of SIRT1 expression in d‐gal‐treated animals without a vitamin D supplementation regimen. Previous studies showed that vitamin D increased SIRT1 expression against H2O2 production (Polidoro et al., 2013). In this study, vitamin D prevented and protected the animals against the effect of d‐gal on SIRT1 expression. Interestingly, the upregulation of SIRT1 was elicited by a low dose of vitamin D in an equal manner using pre‐induction (preventive) and simultaneous (protective) administration regimens. This equipotency is important to note since various studies have shown that preservation of SIRT1 expression is important for neuroprotective, synaptic plasticity in the hippocampus, memory (Abu‐Omar et al., 2018), and heart function during aging (Hsu et al., 2008).

Our study describes, for the first time, the decrease in calstabin2 expression induced by d‐gal, and that this effect is stronger in the heart than in the brain. Also, vitamin D can increase calstabin2 expression against the administration of d‐gal, with higher potency in its preventive than protective effects. In agreement with the sensitivity shown in the two vital organs to the deleterious effects of d‐gal, the prevention of vitamin D was more potent in the heart than in the brain, while the statistical significance of the magnitude of the protective effects was equal in both organs. These distinct patterns are important since various studies showed that, due to its role in releasing Ca2^+^, RYR2 is associated with cardiovascular and nervous disease. On the other hand, ROS seen as signaling molecules are involved in RYR2 modification. Calstabin2, through the stabilization of the closed form of RYR2, prevents calcium leakage into the cytoplasm. As a result, different studies showed that calstabin2 has a role in heart and brain function (Abu‐Omar et al., 2018). It has also been reported that deletion of the calstabin2 gene in mice causes activation of Ca2^+^‐dependent potassium pathways through the increase in intracellular Ca2^+^. This deletion results in hippocampal nerve apoptosis and memory impairment (Yuan, Deng, et al., 2016). Also, previous studies have shown decreased RyR2 expression during aging.

In this study, we studied male mice because d‐gal induces higher oxidative stress in this sex, as we have previously demonstrated. Brains of female mice have been demonstrated to exert lower oxidant and higher antioxidant capacity, but further studies should investigate sex‐dependent differences.

## AUTHOR CONTRIBUTIONS

**Sahar Salemi:** Methodology (equal). **Mohammad Yasin Zamanian:** Conceptualization (equal). **Lydia Giménez‐Llort:** Writing – original draft (equal). **Zahra Jalali:** Methodology (equal). **Mehdi Mahmoodi:** Writing – review and editing (equal). **Maryam Golmohammadi:** Writing – review and editing (equal). **Ayat Kaeidi:** Writing – review and editing (equal). **Zahra Taghipour:** Methodology (equal). **Morteza Khademalhosseini:** Data curation (equal); software (equal). **Mona Modanloo:** Writing – review and editing (equal). **Mohammad Reza Hajizadeh:** Conceptualization (equal); data curation (equal); investigation (equal); project administration (equal); supervision (equal); visualization (equal).

## FUNDING INFORMATION

This work was supported by grant no. 97175 from the Vice Chancellor for Research and Technology, Rafsanjan University of Medical Sciences, Rafsanjan, Iran.

## CONFLICT OF INTEREST STATEMENT

The authors declare no conflict of interest.

## Data Availability

The data that support the findings of this study are available on request from the corresponding author.
